# Examining online postings on a russian internet self-harm message board: Further evidence of addiction to self-harm?

**DOI:** 10.1192/j.eurpsy.2021.1597

**Published:** 2021-08-13

**Authors:** C.A. Lewis, S. Davis, M. Khukhrin, S. Galyautdinova

**Affiliations:** 1 Department Of Social And Behavioural Sciences, School Of Social And Health Sciences, Leeds Trinity University, Leeds, United Kingdom; 2 Psychology, Bashkir State University, Ufa, Russian Federation

**Keywords:** self-harm, Addiction, Russian, online

## Abstract

**Introduction:**

There has been an increasing amount of research examining the addictive nature of self-harm (non-suicidal self-injury). One such area of research has examined if themes related to addiction are present in self-harm board postings on imessages. Recent research from the UK suggests that such themes are evident.

**Objectives:**

The present aim was to build on previous research to examine if themes of addiction are present in other cultural contexts.

**Methods:**

A sample of 254 online postings from a self-harm discussion forum on a Russian Internet message board were translated, extracted, read, and re-read before being coded using inductive content analysis to identify themes.

**Results:**

Five themes were extracted and labelled: “Relationships with Family and Friends”, “Self-Blame and Hatred”, “Ongoing Battle”, “Positive affect”, “Other Mental Health Problems Difficulties”. These themes are somewhat similar to those found within messages in a UK based self-harm forum.

**Conclusions:**

The present findings, obtained from Russian respondents, provide further evidence demonstrating that repetitive self-harming seems to have addictive aspects.

